# Effects of slope and speed of escalator on the dispersion of cough-generated droplets from a passenger

**DOI:** 10.1063/5.0046870

**Published:** 2021-04-02

**Authors:** Zhaobin Li, Xinlei Zhang, Ting Wu, Lixing Zhu, Jianhua Qin, Xiaolei Yang

**Affiliations:** 1 The State Key Laboratory of Nonlinear Mechanics, Institute of Mechanics, Chinese Academy of Sciences, Beijing 100190, China; 2 School of Engineering Sciences, University of Chinese Academy of Sciences, Beijing 100049, China

## Abstract

During the pandemic of COVID-19, the public is encouraged to take stairs or escalators instead of elevators. However, the dispersion of respiratory droplets in these places, featured by slopes and human motion, is not well understood yet. It is consequently unclear whether the commonly recommended social-distancing guidelines are still appropriate in these scenarios. In this work, we analyze the dispersion of cough-generated droplets from a passenger riding an escalator with numerical simulations, focusing on the effects of the slope and speed of the escalator on the droplet dispersion. In the simulations, a one-way coupled Eulerian–Lagrangian approach is adopted, with the air-flow solved using the Reynolds-averaged Navier–Stokes method and the droplets modeled as passive Lagrangian particles. It is found that the slope alters the vertical concentration of the droplets in the passenger's wake significantly. The deflection of cough-generated jet and the wake flow behind the passenger drive the cough-generated droplets upwards when descending an escalator and downwards when ascending, resulting in both higher suspension height and larger spreading range of the viral droplets on a descending escalator than on an ascending one. These findings suggest that the present social-distancing guidelines may be inadequate on descending escalators and need further investigation.

The COVID-19 pandemic is impacting nearly every aspect of society worldwide and is becoming a formidable global public health challenge.^1^ The principal mode by which people are infected with COVID-19 is through exposure to respiratory droplets carrying infectious virus,^2^ which are exhaled from a patient when breathing, talking, coughing, or sneezing.^3–7^ In this process, the viral droplets expelled from the patient are first dispersed in the ambient air flow before finally being inhaled by a susceptible.^8^ For this reason, the droplet spreading is much influenced by the surrounding air flow, which can be complex in some real-world scenarios. Previous studies have advanced our understanding of droplet dispersion in complex environmental flows, by considering the influence of wind,^9^ ventilation systems in classrooms^10–13^ and in restaurants,^14,15^ the use of air conditioners,^16,17^ toilets,^18,19^ and air purifiers in elevators,^20^ and in public transports.^21^ The obstructing effect of face-masks has also been widely explored.^22–27^ Moreover, the droplet dispersion can also be altered by the flow induced by human motion, such as walking,^28^ running, and cycling.^29^ Li *et al.*^28^ showed that human walking can induce a recirculation zone in the wake, which affects the spatial distribution of cough-generated droplets, forming the “attached” mode or the “detached” mode. These different dispersion modes, determined by the space sizes, can result in significantly different contamination regions, suggesting that different safe social-distances are needed for corridors of different widths.

During the pandemic, the public tends to take stairs or escalators more often compared to elevators. However, most existing studies only consider cases on a flat floor, whereas the droplet dispersion on slopes (in stairwells or on escalators), especially when human motion is involved, is not well understood yet, such that it is still unclear if the common social-distancing guidelines^30,31^ are still appropriate on stairs and escalators as epidemiology reports revealed transmission evidence in these places.^32^ To this end, this work investigates the dispersion of cough-generated droplets from a passenger riding an escalator with computational fluid dynamics (CFD), focusing on the influence of escalator's slope and speed on the droplet dispersion with the goal to supplement the current social-distancing guidelines. It is found that the vertical concentrations of viral droplets are very different on ascending escalators and on descending ones, which suggests different social-distancing guidelines are needed for escalators moving in different directions.

The numerical method consists of an one-way coupled Eulerian–Lagrangian approach. In this approach, the air flow around a moving person is first solved in the Eulerian framework and then the obtained flow fields are used by the Lagrangian solver to simulate the transient evolution of the droplets. This one-way coupling is valid for this study because the volume fraction of the cough-generated saliva droplets is often smaller than $10^{-6}$ in the flow around a person,^28,33^ such that the flow can be considered as a dilute particle-laden flow with negligible influence of particles on the flow.^34,35^

The turbulent air flow around the moving person is simulated by the incompressible Reynolds-averaged Navier–Stokes (RANS) equations with Shear Stress Transport (SST) k−ω model,^36^ using the simpleFoam solver of OpenFOAM package.^37^ The dispersion of the respiratory droplets expelled from the person is simulated with the Lagrangian approach, in which the droplets are modeled as passive particles with only three translational degrees of freedom with the rotation and the interactions between droplets neglected. The droplets move with a deterministic velocity computed from the gravity, the buoyancy and air flow's drag forces, plus a stochastic velocity due to turbulence, which is computed with the turbulent dispersion model of Gosman and Ioannides,^38^ as

$$v_{t}^{i}=\sigma \sqrt{\frac{2k}{3}},$$ (1)

with $v_{t}^{i}$ the fluctuation velocity in *i* direction (i∈{x,y,z}), *σ* a random number following the standard normal distribution *N*(0, 1) and *k* the turbulent kinetic energy computed by the flow solver. The droplet dispersion is simulated with OpenFOAM's icoUncoupledKinematicParcelFoam solver. In addition, an evaporation model^39^ is implemented to model the mass and the diameter (*D*) change of droplets as follows:

$$\frac{dD}{dt}=-\frac{4M_{L}D_{v,f}}{D\rho_{L}RT_{f}}\Delta p\left(1+0.276Re^{1/2}Sc^{1/3}\right)\;\;\;\;[m/s],$$ (2)

where *D* is the droplet diameter, *M_L_* is the molecular weight of water (0.018 kg/mole), $D_{v,f}$ is the average diffusion coefficient for vapor molecules in the saturated film around the droplet, $\rho_{L}=998\;\text{kg}/m^{3}$ is the density of water, R=8.3144 J/(mol K) is the gas constant, *T_f_* is the temperature in the saturated film around the droplet, *Re* is Reynolds' number, *Sc* is Schmidt's number, and Δp is the difference between the vapor pressure near the droplet and that in the ambient atmosphere. According to a recent study,^40^ this model can estimate well the evaporation of cough-generated droplets in motion. These parameters are dependent on the environment temperature *T* and relative humidity (*RH*), which are chosen as T=20 °C and RH=50% in this study, representing a winter indoor environment approximately.^41^ The final residual diameter of droplets is set to be 20% the initial diameter according to a recent study on virus-laden saliva droplets.^42^

The cases studied represent daily scenarios where a person is riding an escalator, which is defined by two main characteristics, i.e., the slope *θ* and the moving speed *U* of the escalator. Typically, escalators (or moving walkways) have an inclination angle less than $30^{{}^{\circ}}$, and their operation speed is adjustable in the range 0.40 m/s–0.90 m/s approximately.^43^ The parameter space of this study is shown in Table I. We define ascending escalators with positive slopes and descending escalators with negative slopes. Among these slopes, ±30° are the most common slopes for escalators, while ±15° and 0° are used to represent moving walkways. Among the speeds, 0.4 m/s, 0.65 m/s, and 0.9 m/s are in the operational range and the other two speeds (1.2 m/s, 1.5 m/s) are added to represent passenger's walking. In total, 25 cases are investigated.

**TABLE I.** The parameter space of this study.

Parameter A	Slope-θ [°]	Comment
A-1	−30	Descending escalator
A-2	−15	Descending walkway
A-3	0	Flat walkway
A-4	15	Ascending walkway
A-5	30	Ascending escalator
Parameter B	Speed-*U* [m/s]	Comment
B-1	0.40	Slow operation speed
B-2	0.65	Average operation speed
B-3	0.90	Fast operation speed
B-4	1.20	Passenger walking
B-5	1.50	Passenger fast walking

In the simulations, a human-shape manikin with details of the human body and clothes is employed, as shown in Fig. 1(a). The manikin represents a medium-built male with the height of 1.80 m and the shoulder breadth of 0.45 m. The manikin is assumed to be a rigid body that moves with the escalator. The motion of arms, legs, and other body parts relative to the overall movement is not considered to simplify the problem. The computational domain is rectangular and is rotated according to the slope, as shown in Fig. 1(c). The domain's height is *h* = 3.0 m, the length in front of the manikin is $l_{1}=4.0$ m, and the length behind the manikin is $l_{2}=16.0$ m. The domain half-breadth is set to be 3.0 m with a symmetry *xz* plane, representing a wide open space. The steps of the escalator are replaced with a slope for simplicity. In the flow simulation, the reference frame is fixed to the manikin with air blowing from the inlet. Free-slip conditions are imposed on the lateral and top boundaries. Non-slip conditions are imposed on the manikin and the bottom boundary. The background mesh is Cartesian with Δh=0.1 m. Local mesh refinement are applied near the manikin. The $y^{+}=yu_{\tau }/\nu \approx 1$ for of cells next to the manikin, with *y* the distance to the surface, $u_{\tau }$ the friction velocity, and *ν* the kinematic viscosity (where the friction velocity is determined by the wall shear stress *τ_w_* and the air density *ρ*: $u_{\tau }=\sqrt{\tau_{w}/\rho }$). A wall model based on the logarithmic law^44^ is applied on the bottom boundary, where *y*^+^ is in the range of $\left(35<y^{+}<300\right)$. The total number of cells is approximately 1.3 × 10^6^.

**FIG. 1.**
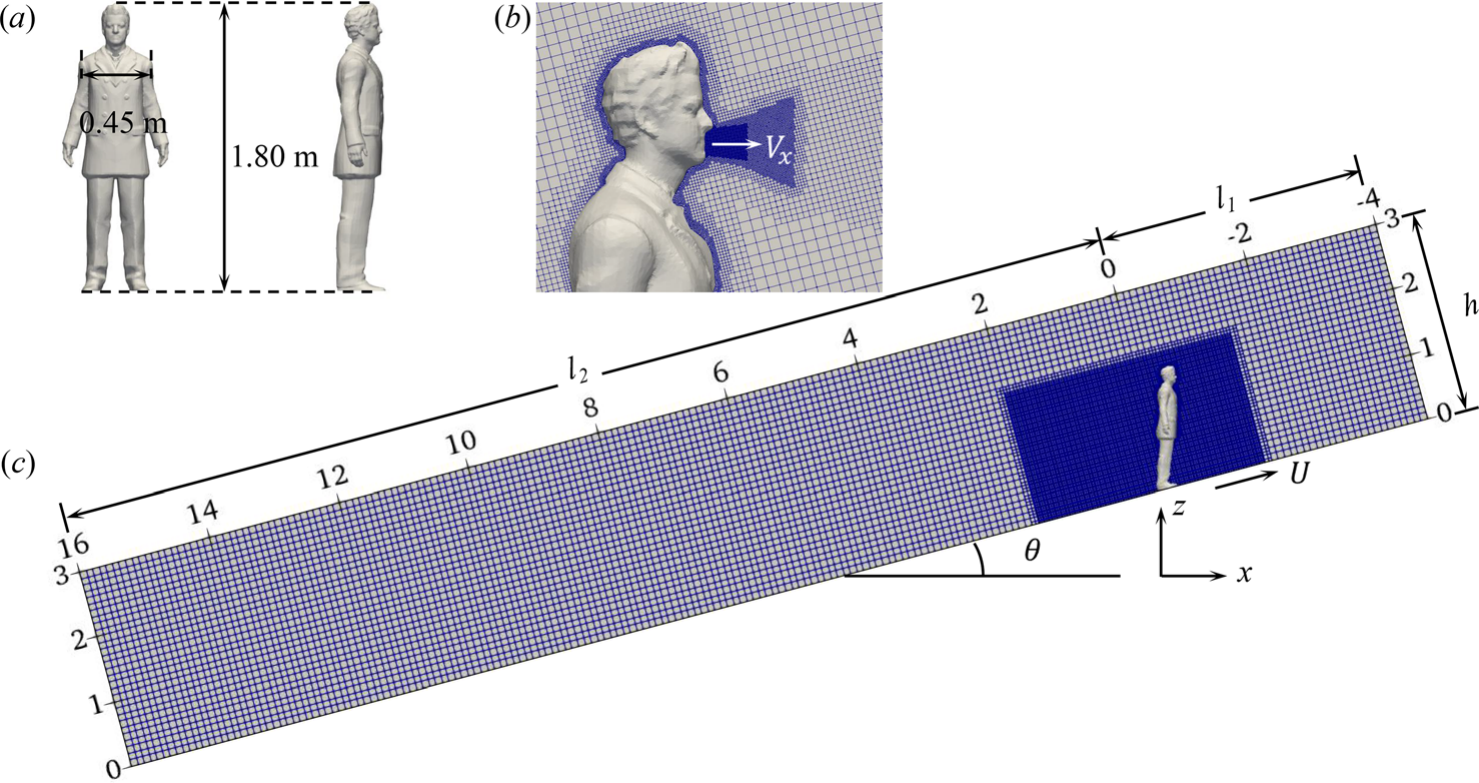
(a) The manikin used in the simulations, (b) close-up on the local mesh refinement near the manikin, and (c) overview of the computational domain and mesh configuration.

This work considers the effect of cough-generated jet-flow on the dispersion of saliva droplets expelled from the mouth. The jet-flow is simplified with a constant horizontal velocity without considering its unsteadiness. Cells are refined accordingly as shown in Fig. 1(b). The droplets are modeled as a cloud of spheroid particles, which are injected into simulations within the first 0.12 s. Following Dbouk and Drikakis,^25^ the droplet diameter *d* follows the Weibull distribution, with d∈[1.0,300.0] *μ*m and $\bar{d}=$ 80.0 *μ*m, where the total number of droplets is 1008 with a total mass of 7.7 mg. The initial velocity of the jet-flow and the particles is set to be $V_{x}=6.5$ m/s according to a recent experiment.^40^ The manikin and the boundaries of the computational domain are assumed to be adhesive so that the droplets reaching these surfaces remain attached. The integration time step is fixed to $\Delta t=5\times 10^{-5}$ s.

To illustrate the effect of the slope on the air flow, Fig. 2 compares the velocity field around the manikin for cases with different inclination angles, i.e., θ=0° and ±30° when the escalator operates at its average velocity of *U* = 0.65 m/s. Velocity components parallel with ($U_{\tau }$) and normal to (*U_n_*) the slope are plotted on the central symmetry plane with streamlines for visualizing principal flow structures. As seen, the manikin decelerates the flow as a bluff body does, forming a recirculation region behind the torso and a wake in the downstream, and the influence of the manikin's legs is relatively small. However, the shape and the extent of the recirculation regions are remarkably different for cases with positive (ascending), negative (descending) and zero (advancing) slopes. The re-circulation region is the smallest in the ascending case [Figs. 2(a) and 2(d)], where the flow is almost attached on the manikin, while the largest re-circulation region is found in the θ=0° case [Figs. 2(c) and 2(f)], where two re-circulation regions are found behind the upper and lower back. Besides, significant difference are observed for the normal velocity components in the manikin's wake. In the ascending case [Fig. 2(d)] a “downwash” velocity component behind the manikin directs the streamlines in the wake toward the slope. In contrast, an “upwash” velocity is found in the descending case [Fig. 2(e)]. For the case without slope [Fig. 2(f)], the directions of *U_n_* are different in the region below and above the waist height where the streamlines converge, being in agreement with Ref. 28. The presence of upwash and downwash velocity in the wake for cases with non-zero slopes can be explained by the inclination of the manikin with respect to the free stream flow, similar to the skewed wakes commonly observed when the obstacle and the inflow direction are misaligned, such as behind an inclined disk^45^ or yawed wind turbines.^46–48^ If making an analogy, the ascending manikin can be regarded as an airfoil with a positive angle of attack (AoA) and the leading edge is located at the head and the shoulder. The airfoil receives an upward force from the air flow and thus the reaction force creates a “downwash” in the wake.^49^ In the descending case, leading edge is the lower torso and the body resembles an airfoil of negative AoA, which generates an “upwash” in the wake. In addition to the influence on the wake, the slope is also found altering the cough-generated jet from the mouth. The jet is redirected slightly downwards in the ascending case as shown in Figs. 2(a) and 2(d) and upwards in the descending case as shown in Figs. 2(b) and 2(e). This behavior is similar to jets in crossflow.^50^ It is worth noting that the jet for the case without slope [Figs. 2(c) and 2(f)] also turns up slightly, because the air flows upwards slightly in front the manikin's head.

**FIG. 2.**
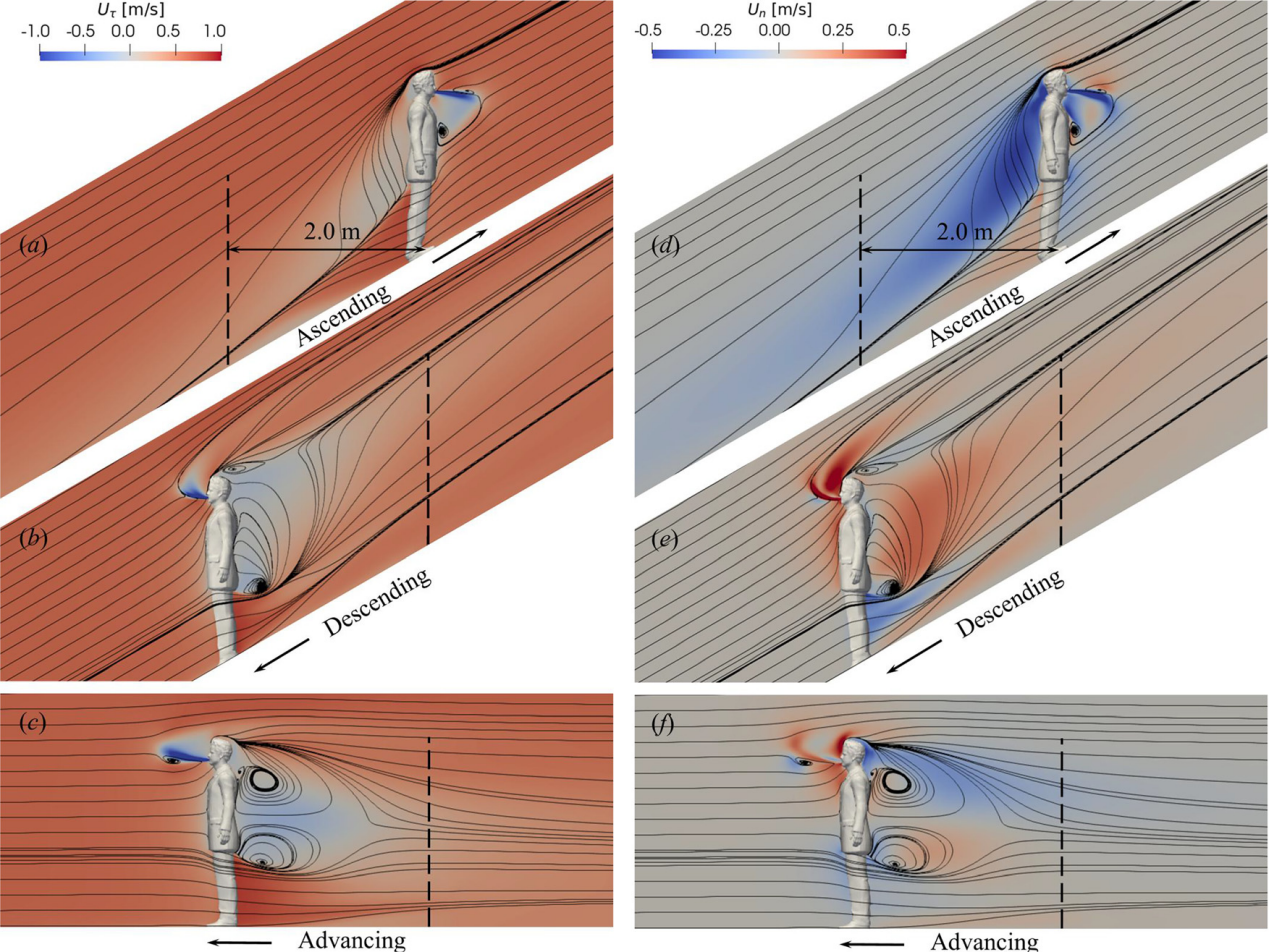
Air flow around the passenger at speed *U* = 0.65 m/s on (a) and (d) an ascending escalator with slope θ=30° and (b) and (e) a descending escalator with slope θ=−30°, (c) and (f) a moving walkway with θ=0°. (a)–(c) and (d)–(f) show the velocity component parallel with and normal to the slope, respectively.

The obtained flow fields are then employed in simulating the dispersion of the cough-generated droplets. Figure 3 (Multimedia view) compares the droplet evolution on escalators with the same advancing speed *U* = 0.65 m/s but different slopes (−30°,  0°,  30°). The initial behavior of the droplet cloud expelled from the manikin's mouth, marked as red dots, is similar for cases with different slopes. However, at *t* = 2.0 s, the influence of the slope starts to appear: the droplets in the ascending case are clustered in front of the upper body of the manikin, whereas the droplets in the descending and the flat floor case are convected over and around the manikin's head. This vertical difference is related to the cough-generated jet, which is skewed downwards in the ascending case and upwards in the descending case as shown in Fig. 2. Furthermore, the ascending case also shows slower horizontal particle motion in downstream direction, suggesting that the torso has larger obstruction effect compared to the head. At t=4.0 s, the effects of the wake flow on the droplets dispersion behind the passenger are clearly illustrated with the streamlines. In the ascending case, the droplets are convected downwards due to the “downwash” in the wake, whereas the “upwash” in the descending case blows the droplets upwards. Moreover, droplets are entrapped by the re-circulation bubble behind the manikin for the case with θ=0°, where the majority of the droplets falls into the re-circulation bubble forming a typical “attached” dispersion mode as defined in Ref. 28. For the case with θ=−30°, approximately a half of the droplets are entrapped, and the rest droplets are escaped from the re-circulation region. In contrast, no apparent droplet entrapment is observed in the ascending case as the re-circulation bubble is very small. Finally, these differences in flow structures lead to distinctively different droplet dispersion patterns. For the ascending case, the result at *t* = 8.0 s shows that the droplets are slightly detached from the coughing passenger and the height of the droplet cloud is very low. These droplets contaminate only the legs of an adult if he/she stands two meters behind, suggesting a relatively low transmission risk. However, a follower standing two meters behind the coughing passenger is exposed to a much higher risk on a descending escalator, because the viral-laden droplets float upwards and concentrate at the head height of an adult, making them much easier to be inhaled by the susceptible. At *t* = 8.0 s, the number of droplets suspending 1.5 m higher from the ground is 2, 188, 500, for the θ=30°,0°,−30° cases, respectively, out of 1008 in total. This height difference in the contamination region due to slope is also observed in cases with other moving speeds. Figure 4 shows an example at *U* = 1.5 m/s with slopes θ=±30° at *t* = 8.0 s. In Fig. 4, the rulers show the distance from the coughing position x′=0. In Fig. 4(a), only a small number of droplets can be observed close to the ground at x′≈4 m, other droplets either fall onto the manikin's chest in the front or fall onto the escalator due to the gravity and the “downwash” in the wake. In contrast, much more droplets remain suspended in the range of x′∈[0,6] m in the descending case, because less droplets are attached on the manikin in the beginning stage and because the settling of droplets is delayed due to the “upwash” in the wake. In both cases, the droplets leave away from the coughing passenger, forming a “detached mode” as defined by Li *et al.*^28^ In the descending case, the droplet cloud is still suspending highly in the air even the passenger have moved more than ten meters in front, suggesting that fast descending on escalator can extend drastically the spreading range of the droplets.

**FIG. 3.**
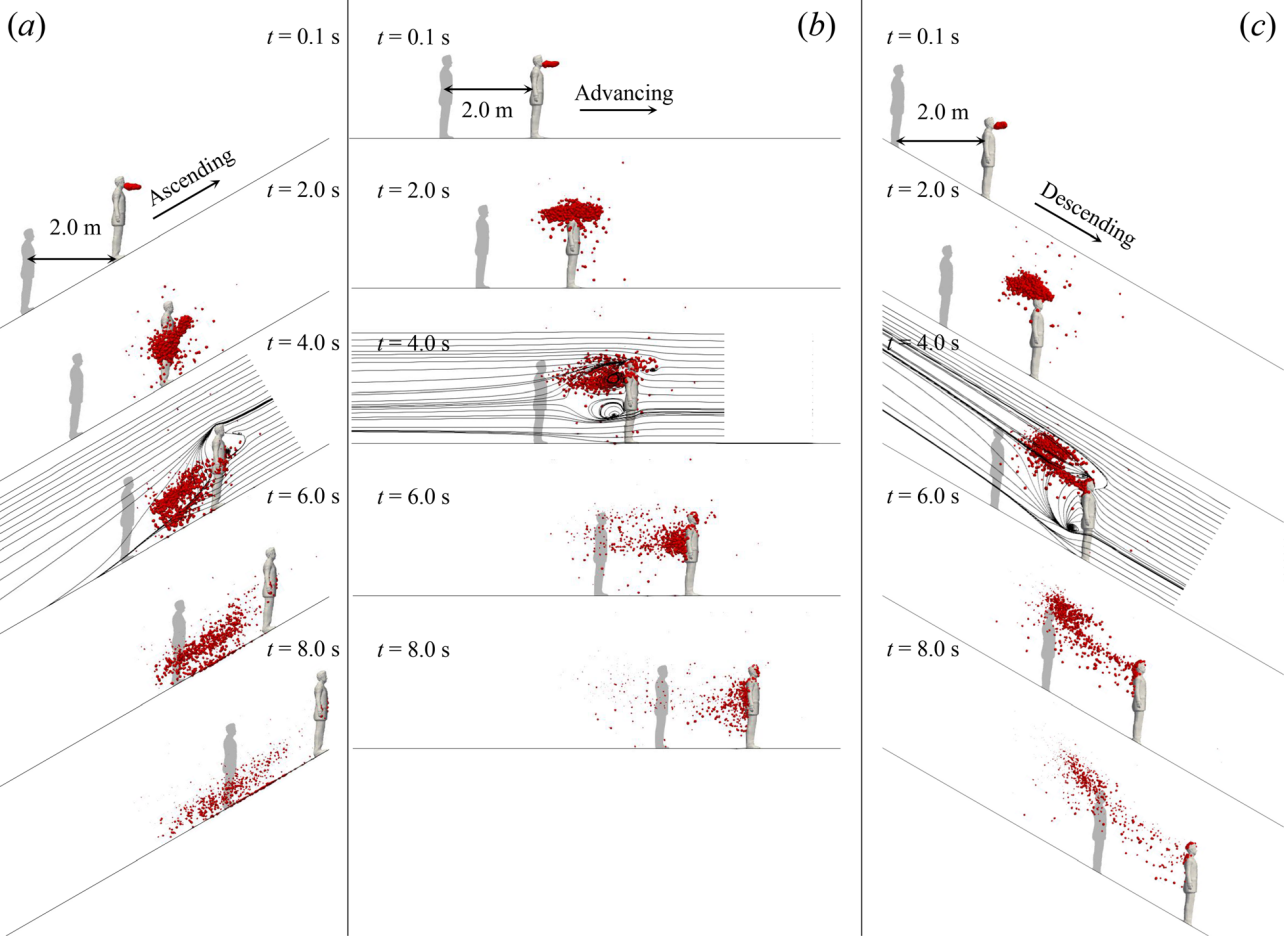
Patterns of droplet dispersion in the wake of the manikin for: (a) ascending escalator with slope θ=30°, (b) moving walkway without slope, and (c) descending escalator with slope θ=−30°. The speed is 0.65 m/s, representing the normal operation condition. Streamlines are added to illustrate flow structures at t=4.0 s. The shadow manikins illustrate the 2-m social-distance. Droplet diameters are scaled-up 1000 times. Multimedia view: https://doi.org/10.1063/5.0046870.1
10.1063/5.0046870.1

**FIG. 4.**
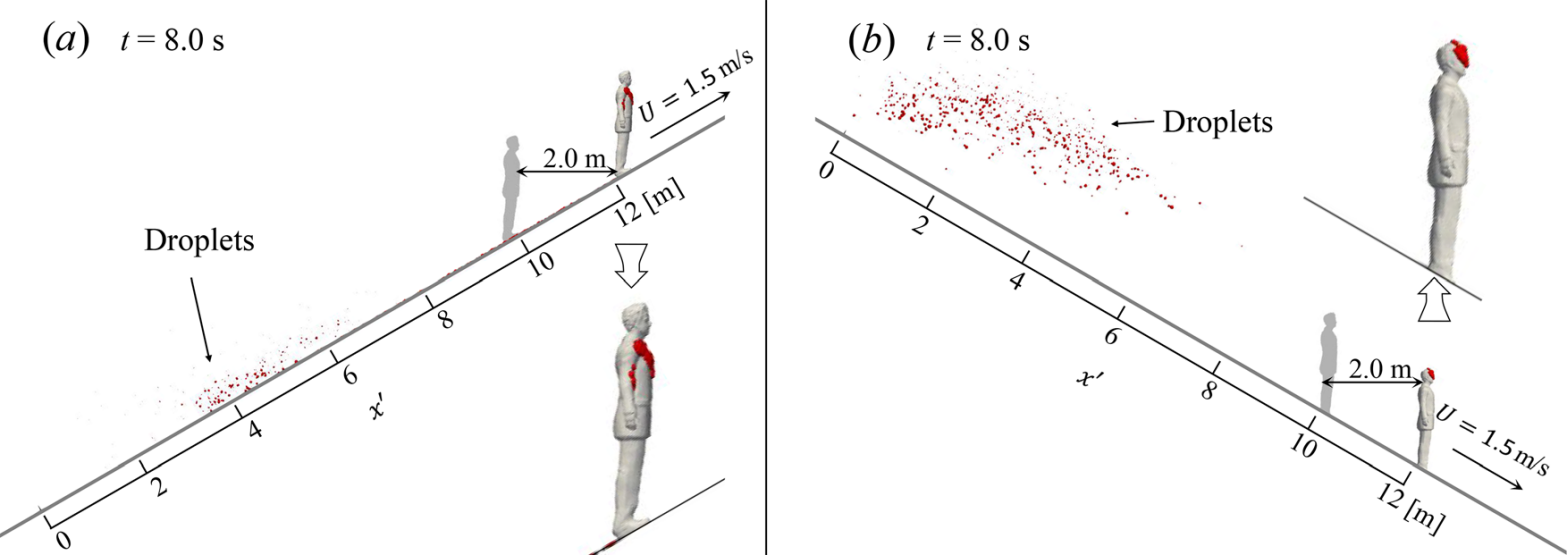
Patterns of droplet dispersion in the wake of the manikin moving on escalators with slope (a) θ=30° and (b) θ=30° and speed *U* = 1.5 m/s at *t* = 8.0 s. Droplet diameters are scaled-up 1000 times.

The vertical concentration of droplets is then further analyzed with the 25 simulations from our parameter spaces defined in Table I. Besides the “attached” and “detached” modes from Li *et al.*^28^ describing the horizontal distribution, we define three extra vertical modes, referred to as “lower”, “middle” and “upper” for the vertical locations of the droplet cloud, which correspond to the scenarios illustrated in Figs. 3(a)–3(c), respectively. Figure 5 shows a mode map for different slopes *θ* and different speeds *U*. The “lower” mode is found in all ascending cases, whereas the “upper” mode appears only for cases with the steepest negative slope (θ=−30°) and with adequately fast moving speed (U≥0.65 m/s), i.e., when the “upwash” in the wake is strong. Besides, the detached mode are observed in cases with relative slow moving speed on an ascending escalator, because the re-circulation bubble behind the manikin is small in ascending cases.

**FIG. 5.**
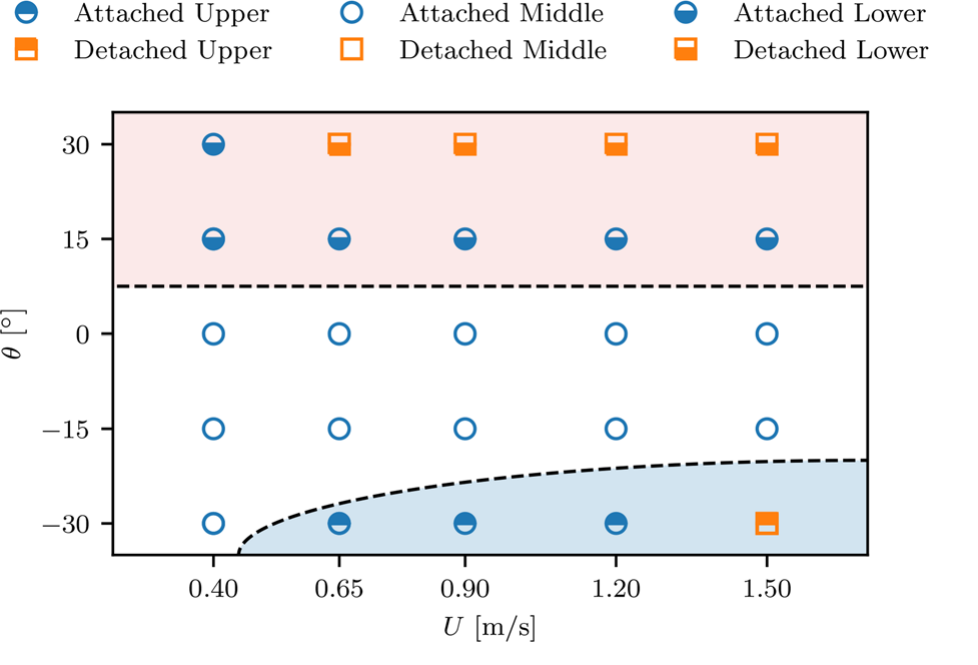
The mode map in the parameter space of the slope *θ* and the speed *U*.

In summary, we investigated the effects of slope and speed on the dispersion of cough-generated droplets behind a passenger riding an escalator with a one-way coupled Eulerian–Lagrangian method. The flow structures around and behind the passenger are found being significantly different when the slope direction is altered. The cough-generated jet is skewed downwards in the ascending case and upwards in the descending case. Besides, we observe a “downwash” flow in the passenger's wake for cases with ascending slopes and an “upwash” flow for cases with descending slopes. More importantly, these flow structures lead to three droplet dispersion modes in the vertical concentration, i.e., (1) the “middle” mode, representing the ordinary vertical dispersion in the passengers wake, which has been observed in Li *et al.*;^28^ (2) the “lower” mode, occurring for cases with ascending slopes, in which the droplets are clustered close to the ground helping to reduce the transmission risk; and (3) the “upper” mode, happening on a descending escalator, in which the suspension of viral droplets appears at approximately the head height of adult and over a long horizontal range, which indicates higher risk to inhale these viral droplets for passengers riding a descending escalator. Moreover, the viral droplet spreading range is found to be further extended if the passenger walks on a descending escalator. These results suggest that the commonly practiced social-distancing,^30,31^ which may be considered as adequate for ascending escalators, is not appropriate for descending ones from the fluid dynamics point of view and needs to be re-designed. This study suggests that farther distance should be recommended for people riding descending escalators and fast walking on escalators must be avoided.

## Data Availability

The data that support the findings of this study are available from the corresponding author upon reasonable request.
